# A Method for Evaluating Dynamical Friction in Linear Ball Bearings

**DOI:** 10.3390/s101110069

**Published:** 2010-11-09

**Authors:** Yusaku Fujii, Koichi Maru, Tao Jin, Preecha P. Yupapin, Somsak Mitatha

**Affiliations:** 1 Department of Electronic Engineering, Faculty of Engineering, Gunma University, 1-5-1 Tenjin-cho, Kiryu 376-8515, Japan; E-Mail: k.billow@gmail.com (T.J.); 2 Advanced Research Center for Photonics, King Mongkut’s Institute of Technology Ladkrabang Chalongkrung Road, Ladkrabang, Bangkok 10520, Thailand; E-Mail: kypreech@knitl.ac.th (P.P.Y.); 3 Department of Computer Engineering, King Mongkut’s Institute of Technology Ladkrabang, Chalongkrung Road, Ladkrabang, Bangkok 10520, Thailand

**Keywords:** linear ball bearings, dynamic friction coefficient, inertial force, optical measurement

## Abstract

A method is proposed for evaluating the dynamical friction of linear bearings, whose motion is not perfectly linear due to some play in its internal mechanism. In this method, the moving part of a linear bearing is made to move freely, and the force acting on the moving part is measured as the inertial force given by the product of its mass and the acceleration of its centre of gravity. To evaluate the acceleration of its centre of gravity, the acceleration of two different points on it is measured using a dual-axis optical interferometer.

## 1. Introduction

The important characteristics of bearings are precise motion and small friction. The friction of the bearing is of great interest in the field of precision engineering [1–4]. However, techniques for measuring the friction of linear bearings have not been sufficiently investigated. Conventional techniques such as the technique using a force transducer [1] and that using the gravitational force acting on a weight [2–4] do not provide sufficient precision for some applications. Considering the important role of linear bearings, it is essential to develop techniques for evaluating their frictional characteristics with high accuracy.

To measure the instantaneous value of the frictional force in motion acting inside the linear bearings, force transducers are usually used. However, the force transducers are typically calibrated by standard static methods using static weights under static conditions. At present, there are no standard methods for evaluating the dynamic characteristics of force transducers. This leads to two major problems in material tester using force transducers: (1) it is difficult to evaluate the uncertainty in the measured varying force and (2) it is difficult to evaluate the uncertainty of the instant at which the varying force is measured.

The first author has proposed a precision force measurement method, the Levitation Mass Method (LMM). In this method, the inertial force of a mass levitated by means of a pneumatic linear bearing is used as the reference force and this force is applied to the objects being tested, such as force transducers, materials or structures. The inertial force of the levitated mass is measured as the product of the mass and acceleration. In the LMM, only the Doppler shift frequency of the laser beam reflected from the levitated object is measured using an optical interferometer. All the other quantities such as velocity, position, acceleration and inertial force are calculated from the measured frequency.

The author has modified the LMM into a dynamic method for the calibration of force transducers, such as the dynamic calibration method for oscillation loads [5] and the method for correcting the effect of the inertial mass of the transducers [6]. Apart from the LMM, Kumme has also proposed and developed a method, in which the inertial force of a mass directly attached to a force transducer is used [7,8]. In this method, both the mass and the transducer are shaken at a single frequency using an electromagnetic shaker, and the inertial force of the mass is applied to the transducer. Park *et al*. used this method for dynamic investigation of multi component force-moment sensors [9,10].

However, it is not yet known how the results of such dynamic calibration can be applied to the actual wave profile of a varying force. This difficulty is mainly due to the fact that the validity of employing the frequency response obtained from the oscillation force calibration for other types of forces such as the impact force has not been proven, therefore the frequency response is unlikely to be used for other types of forces.

On the other hand, some methods of analyzing the frequency response of force transducers under free oscillating condition have been proposed [11,12]. In these methods, the zero-value force under free oscillation condition is used as the reference force. Since the reference force is zero, these methods cannot be considered to be dynamic calibration methods for force transducers.

The first author has employed the LMM for material testing, e.g., in a method for evaluating the material viscoelasticity [13] and in methods for generating and measuring micro-Newton level forces [14,15]. The author has also employed the LMM for investigating the frictional characteristics of pneumatic linear bearings [16,17]. The author has also used it for investigating the frictional characteristics of linear ball bearings, whose motion is almost linear with negligible pitching vibration [18]. In such methods, no force transducers are used and the force is directly measured based on the definition of force, *i.e.*, the product of mass and acceleration. However, it is still difficult to evaluate the dynamical friction acting on a usual linear ball bearing, whose motion is not perfectly linear due to some play, which is slack or gap between the moving part and the guideway. The play causes the translational and rotational vibrations of the moving part. The reasons for this difficulty are as follows:

1. The acceleration is measured at the measurement point, *i.e.*, at the optical center of the cube-corner prism.
2. The measurement point and center of gravity of the moving part are separated.
3. The vibration of the moving part due to the slack or play results in a change in the difference between the acceleration of the measurement point and that of the center of gravity of the moving part.

In this paper, a novel method for overcoming the above difficulties due to the pitching vibration of the moving part of the bearing is proposed. In the proposed method, the accelerations of two different points on the moving part are measured using a dual-axis optical interferometer. The relative positions between the two measurement points and the center of gravity are evaluated beforehand using the balancing method. The acceleration of the center of gravity is calculated from the accelerations of two measurement points.

## 2. Experimental Setup

**Figure 1.**
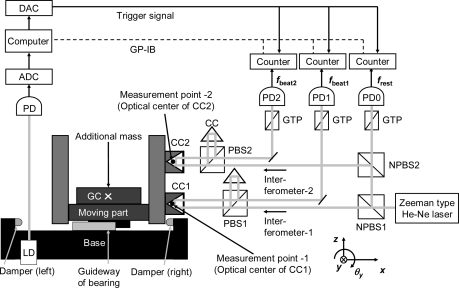
Experimental Setup. Code: GC = center of gravity, CC = cube corner prism, PBS = polarizing beam splitter, NPBS = non-polarizing beam splitter, GTP = Glan-Thompson prism, LD = laser diode, PD = photo diode, PC = computer, ADC = analog-to-digital converter, DAC = digital-to-analog converter.

**Figure 2.**
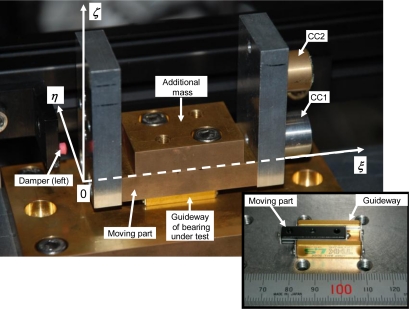
Photographs of the bearing under testing, in which the coordinate system is shown.

Figure 1 shows the experimental setup used for evaluating the frictional characteristics of a linear ball bearing with some play in the internal mechanism. Figure 2 shows a photograph depicting the region around the test section.

Two cube-corner prisms CC1 and CC2 are attached to the moving part of the bearing. The coordinate system fixed in space (x, y, z) is set as shown in Figure 1. The coordinate system fixed to the moving part (ξ, η, ζ) is set as shown in Figure 2. The relative positions of the optical centers of CC1 and CC2 are ***P*_CC1_** = (ξ**_CC1_**, η**_CC1_**, ζ**_CC1_**) = (75.2 mm, 0.0 mm, 12.0 mm) and ***P*_CC2_** = (ξ**_CC2_**, η**_CC2_**, ζ**_CC2_**) = (74.7 mm, 0.0 mm, 37.7 mm), respectively. The measurement points are ***P*_CC1_** and ***P*_CC2_**. The height difference ***L*** between the optical centers of CC1 and CC2 is approximately ***L*** = |ζ**_CC2_** − ζ**_CC1_**| = 25.7 mm.

If the motion of the moving part is perfectly parallel translation, then the accelerations of ***P*_CC1_** and ***P*_CC2_** are equal to the acceleration of the center of gravity (GC) of the moving part, ***P*_GC_**. However, if the motion is not parallel translation, then its rotational motion results in the difference of the accelerations of ***P*_CC1_**, ***P*_CC2_** and ***P*_GC_**. In the proposed method, the acceleration of the center of gravity and the total force acting on the moving part are estimated as follows:

First, ***P*_GC_** is estimated by the balancing method [19] as the following steps. Here, the relative position of the center of gravity (GC) of the moving part, ***P*_GC_**, is thought to be in the ξζ plane since the moving part is symmetrical with respect to the ξζ plane. ***P*_CC1_** and ***P*_CC2_** are also in the ξζ plane. Therefore, the differences between the accelerations of ***P*_CC1_, *P*_CC2_** and ***P*_GC_** are mainly caused by the rotational motion along y-axis, *i.e.*, the pitching motion.

(i). The moving part is separated from the other parts and it is hung using a fine thread at three different points. When the moving part is suspended by a thread from a point, it is in equilibrium under the action of the tension in the thread and the resultant of the gravitational forces of the moving part. When the moving part is suspended from another point, it is again in equilibrium.

(ii). Side-view pictures are taken from the η-axis direction, and the straight lines are marked over the thread on these pictures. These lines indicate the lines of action of the resultant of the gravitational forces. These lines would be concurrent at ***P*_GC_**.

(iii). From the three pictures, ***P*_GC_** is estimated by means of the least squares method.

***P*_GC_** is estimated to be (ξ**_GC_**, η**_GC_**, ζ**_GC_**) = (36.9 mm, 0.0 mm, 15.8 mm) when one additional mass is attached to the moving part. The height of CC1 is closer to that of the GC as compared to the height of CC2.

Second, the acceleration at ***P*_GC_** in the direction of motion, *i.e.*, direction of x-axis, is estimated using the accelerations at the two measurement points, *i.e*., the optical centers of CC1 and CC2:

(1) $$a_{\mathrm{GC}}=(\left(\zeta_{\mathrm{CC}2}-\zeta_{\mathrm{GC}}\right)a_{1}+\left(\zeta_{\mathrm{GC}}-\zeta_{\mathrm{CC}1}\right)a_{2})/\left(\zeta_{\mathrm{CC}2}-\zeta_{\mathrm{CC}1}\right)$$

where ***a*_1_** and ***a*_2_** are the accelerations along x-axis at the optical centers of CC1 and CC2, respectively. Then, the total force acting on the moving part, ***F***, is calculated as the product of the mass of the moving part, ***M***, and the acceleration at the GC, ***a*_GC_**, as

(2) $$F={\mathrm{Ma}}_{\mathrm{GC}}$$

If only one cube-corner prism is used and the height difference between the cube-corner prism and GC is not zero, the acceleration at the cube-corner prism along the x-axis would be sensitive to the rotation along the y-axis, *i.e*., the pitching motion, because the pitching motion easily causes the displacement of the cube-corner prism along the x-axis as the sine error. This causes the measurement error in the acceleration at the GC. Thus, if only one cube-corner prism is used, the heights of the optical center of the prism and the optical setup should be carefully adjusted to be the same as the height of the GC. In the proposed method, on the other hand, the effect of pitching motion can be corrected by Equation (1).

As for the effects along the other axes, their effects on the measurement error are thought to be negligible. More specifically, the acceleration along the x-axis is not affected by the rotation along the x-axis, *i.e.*, the rolling motion, at all. It is weekly sensitive to the rotation along the z-axis, *i.e*., the yawing motion, since that is the cosine error.

A Zeeman-type two-frequency He-Ne laser is used as the light source of the dual-axis optical interferometer. The interferometer has three photo-detectors: PD0, PD1 and PD2. The frequency difference between the two orthogonal polarisation states emitted from the laser, ***f*_rest_**, is monitored using a Glan-Thompson prism (GTP) and the first photo-detector (PD0).

The velocity of CC1, ***v*_1_**, is measured as the Doppler shift frequency ***f*_Doppler1_**, which can be expressed as follows:

(3) $$v_{1}=\lambda_{\mathrm{air}}\left(f_{\mathrm{Doppler}1}\right)/2$$

(4) $$f_{\mathrm{Doppler}1}=–\left(f_{\mathrm{beat}1}–f_{\mathrm{rest}}\right)$$

where ***λ*_air_** is the wavelength of the signal beam under the experimental conditions and ***f*_beat1_** is the beat frequency, which is the frequency difference between the signal beam and the reference beam, that appears at PD1. In the same way, the velocity of CC2, ***v*_2_**, is measured as the frequency Doppler shift ***f*_Doppler2_**.

The frequency ***f*_rest_** appearing at PD0 is measured using an electric frequency counter (model: R5363; manufactured by Advantest Corp., Japan). It continuously measures and records the rest frequency ***f*_rest_** 2,000 times at a sampling interval of T = 4,000/***f*_rest_** and stores the values in its memory. This counter continuously measures the interval time every 4,000 periods without dead time. The sampling period of the counter is approximately 1.4 ms at a frequency of 2.8 MHz. Two other counters of the same model measure the frequencies ***f*_beat1_** and ***f*_beat2_** appearing at PD1 and PD2, respectively. The counters measure the frequencies without dead time, and ***T*** can be exactly calculated using the measured frequency ***f*** and the expression ***T*** = 4,000/***f***. The position ***x*** is calculated by integrating the velocity ***v***. The acceleration ***a*** is calculated by differentiating the velocity ***v***.

The measurements using the three electric counters (R5363) are triggered by means of a sharp trigger signal generated using a digital-to-analog converter (DAC). This signal is initiated by means of a light switch, which is a combination of a laser diode and a photo-diode. In the experiment, only one additional mass is attached to the moving part. The total mass of the moving part with the additional mass ***M*** is 0.311 kg.

## 3. Results

**Figure 3.**
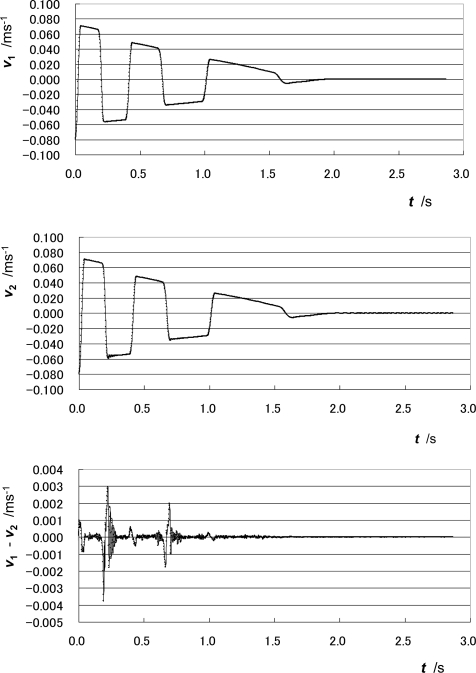
Change in velocities at CC1 and CC2.

Figure 3 shows the change in velocity at CC1, ***v*_1_**, velocity at CC2, ***v*_2_**, and those difference, ***v*_1_** − ***v*_2_**.

During the measurement of approximately 3 seconds, the moving part performs reciprocating motion. It collides with the damper on the left three times and that on the right three times. Relatively large vibrations in the difference between the velocities at CC1 and CC2, ***v*_1_** − ***v*_2_**, are observed after the collision with the dampers, especially with the right damper. These differences are thought to come from the play between the moving part and the guideway of the bearing.

**Figure 4.**
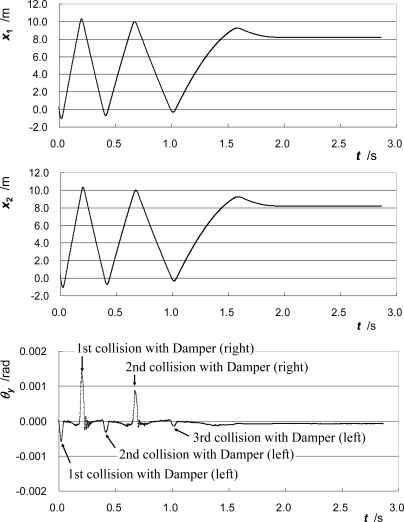
Change in positions at CC1 and CC2 and in pitching angle.

Figure 4 shows the change in position at CC1, ***x*_1_**, the position at CC2, ***x*_2_**, and the pitching angle of the moving part, ***θ_y_***. The positions ***x*_1_** and ***x*_2_** are calculated by integrating the velocities ***v*_1_** and ***v*_2_**, respectively. The pitching angle ***θ_y_*** is calculated using the following expression:

(5) $$\theta_{y}=\delta x/L$$

(6) $$\delta x=x_{\mathrm{CC}2}-x_{\mathrm{CC}1}$$

where ***L*** is the height difference between the optical centers of CC1 and CC2 (***L*** = |ζ**_CC2_** – ζ**_CC1_**|).

The pitching angle ***θ_y_*** is the rotation angle around y-axis, and it changes significantly when the moving part collides with the side dampers. It appears that the slack or play of the bearing around the right side is larger than that around the left side.

**Figure 5.**
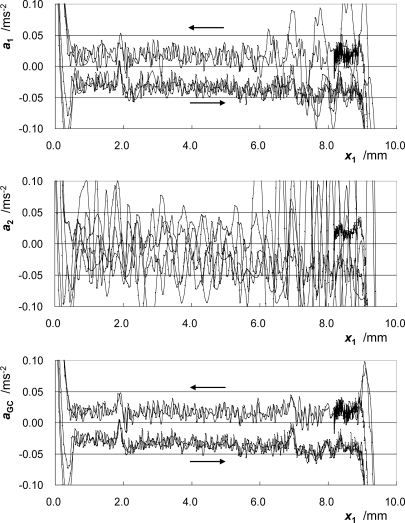
Change in accelerations at CC1, CC2, and GC.

Figure 5 shows the change in acceleration at CC1, ***a*_1_**, the acceleration at CC2, ***a*_2_**, and the acceleration at the GC, ***a*_GC_**. The accelerations ***a*_1_** and ***a*_2_** are calculated by differentiating the velocities ***v*_1_** and ***v*_2_**, respectively. The parameter ***a*_GC_**, *i.e.*, the acceleration at ***P*_GC_** in the direction of motion, is calculated from ***a*_1_** and ***a*_2_** using Eq. (1). In each figure, the three runs of the rightward motion and the two runs of the leftward motion are drawn. The acceleration of CC2, whose height differs considerably from that of the GC as compared to the height of CC1, varies considerably. On the other hand, the plotted lines of ***a*_GC_** in different runs of the moving part coincide well with each other.

**Figure 6.**
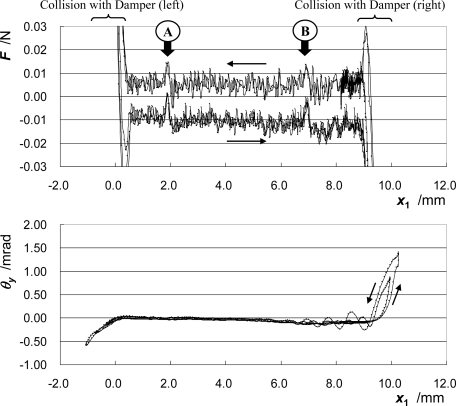
Change in total force acting on the moving part and pitching angle of the moving part against position.

Figure 6 shows the change in the total force acting on the moving part ***F*** and the pitching angle of the moving part ***θ_y_*** against its position. The total force acting on the moving part ***F*** is calculated as the product of the mass of the moving part ***M*** and the acceleration at the GC ***a*_GC_**. In the figure of ***F***, lines of different passes coincide well with each other. Around the regions indicated as A and B in the figure, the total force acting on the moving part has a unique feature. These indicate both the high reproducibility of the frictional force acting inside the bearing and the high accuracy of the measurement.

## 4. Uncertainty Evaluation

The uncertainty components in determining the instantaneous value of the acceleration at the center of gravity of the moving part ***a*_GC_** are as follows:

(a) Measurement of the accelerations ***a*_1_** and ***a*_2_**

The dominant uncertainty source in the accelerations ***a*1** and ***a*2** is the uncertainty in the frequency measurement using the electric frequency counter R5363 of approximately 3 Hz, since the other uncertainty sources, such as the laser alignment, refractive index of air and the wavelength of the laser, are negligible. This corresponds to the uncertainty of the accelerations ***a*_1_** and ***a*_2_** of approximately 2 × 10^−3^ m/s^2^. This results in the standard uncertainty of ***a*_GC_** due to the uncertainty of the accelerations ***a*_1_** and ***a*_2_** of approximately 3 × 10^−3^ m/s^2^.

(b) Estimation of ***P*_GC_**

The uncertainty in estimating the position of the center of gravity of the moving part ***P*_GC_** is estimated to be approximately 0.1 mm. This corresponds to the standard uncertainty of ***a*_GC_** due to the uncertainty of ***P*_GC_** of approximately 0.4 × 10^−3^ m/s^2^.

Therefore, the standard uncertainty in estimating ***a*_GC_** is estimated to be approximately 3 × 10^−3^ m/s^2^. This corresponds to the standard uncertainty in estimating the total force acting on the moving part of approximately 0.9 × 10^−3^ N.

## 5. Discussion

The plotted lines in different runs of the moving part coincide well each other. The coincidence suggests that the frictional force is measured with high reproducibility even if the velocity of the moving part is changed. Thus, using the proposed method, the frictional characteristics of linear bearings with some play in their mechanism can be accurately evaluated. Conventional linear ball bearings have some play, and therefore the proposed method will contribute significantly to research on linear ball bearings and the linear ball bearing industry.

The uncertainty of the method has not been completely evaluated, but the authors estimate that the relative standard uncertainty in measuring the frictional force is approximately 5% in the described experiment. Using the proposed method, the relationships between the time, velocity, acceleration, inertial force, position and pitching angle are accurately measured. Their small changes in the short period can be observed. This will help in understanding the phenomenon and mechanism of frictional force acting inside the linear ball bearing. In the proposed method, only the time-varying frequency is measured during the sliding experiment. The velocity, acceleration, inertial force and position are all calculated from the measured time-varying beat frequency. This induces the synchronisation between the calculated physical quantities and the simplicity of the measurement system.

The effect of pitching motion correction with the proposed method on the improvement in the measurement accuracy is significant, as shown in Figure 5. This fact also indicates that if only one cube-corner prism can be attached to the moving part of the bearing, then the height of the optical center of the prism should be carefully adjusted to be the same as the height of its GC. This adjustment is severe and troublesome especially when the measurement is done for various additional masses. Moreover, the pitching angle cannot be monitored if only one cube-corner prism is used. On the other hand, in the proposed method, the adjustment is not severe and no changes of the height of the optical center of each prism and the optical setup are required even when the additional mass is changed.

In the proposed method, the total mass of the moving part and the relative positions between the two measurement points and the center of gravity should be measured beforehand. Once they are measured under a certain condition of attached masses, then they can be calculated using the mass and the center of gravity of the additional attached mass. If the shape and the density distribution of the moving part is enough known precisely, then the relative positions between the two measurement points and the center of gravity can be numerically calculated. In this case, no measurement of the center of gravity is necessary.

In the LMM, the measurement of frequency is essential. To improve the sampling interval and the resolution of frequency measurement, the introduction of the novel method [20,21] using a digitiser instead of an electric counter will be effective.

## 6. Concluding Remarks

A novel method for evaluating the frictional characteristics of linear bearings with some play in its mechanism was developed by modifying the Levitation Mass Method (LMM). In the measurement, the moving part of a linear ball bearing was made to move freely, and the force acting on the moving part was measured as the inertial force given by the product of its mass and the acceleration of its center of gravity. To evaluate the acceleration of its center of gravity, the acceleration of two different points on it were measured using a dual-axis optical interferometer. The relative positions between the two measurement points and the center of gravity were evaluated beforehand. The measured results indicated the high reproducibility of the frictional force acting inside the bearing and the high accuracy of the measurement. The precision measurements of the frictional characteristics of the linear ball bearings, which are widely used in many applications of mechatronics and robotics, will help in understanding the mechanics of friction and in developing an improved method for the position control of linear actuators with linear bearings. The proposed method will contribute significantly to research on linear ball bearings and the linear ball bearing industry.
